# Phase-1 study of vamotinib (PF-114), a 3rd generation *BCR::ABL1* tyrosine kinase-inhibitor, in chronic myeloid leukaemia

**DOI:** 10.1007/s00277-025-06239-8

**Published:** 2025-04-29

**Authors:** Anna Turkina, Olga Vinogradova, Elza Lomaia, Evgeniya Shatokhina, Oleg Shukhov, Ekaterina Chelysheva, Dzhariyat Shikhbabaeva, Irina Nemchenko, Anna Petrova, Anastasiya Bykova, Nadiya Siordiya, Vasily Shuvaev, Ilya Mikhailov, Fedor Novikov, Veronika Shulgina, Andreas Hochhaus, Oliver Ottmann, Jorge Cortes, Robert Peter Gale, Ghermes Chilov

**Affiliations:** 1 National Medical Research Center for Haematology, Moscow, Russian Federation; 2 Botkin Hospital, Moscow, Russian Federation; 3 Rogachev National Medical Research Center for Pediatric Hematology, Oncology and Immunology, Moscow, Russia; 4 Almazov National Medical Research Centre, Saint-Petersburg, Russian Federation; 5 Medical Research and Educational Center, Lomonosov Moscow State University, Moscow, Russia; 6 A.Tsyb Medical Radiological Research Center, Obninsk, Russian Federation; 7 Russian Medical Academy of Postgraduate Education, Moscow, Russia; 8 Center for Healthcare Quality Assessment and Control of the Ministry of Health of the Russian Federation, Moscow, Russia; 9 Zelinsky Institute of Organic Chemistry, Russian Academy of Sciences, Moscow, Russia; 10 City Clinical Hospital №31, Moscow, Russian Federation; 11 Hematology/Oncology, University Hospital Jena, Jena, Germany; 12 School of Medicine, Cardiff University, Cardiff, UK; 13 Georgia Cancer Center, Augusta University, Augusta, GA USA; 14 Centre for Haematology, Department of Immunology and Inflammation, Imperial College London, London, UK; 15 Fusion Pharma, Nobelya st. 5, Moscow, 121205 Russian Federation

**Keywords:** Chronic myeloid leukaemia, Tyrosine kinase inhibitor, PF-114, Vamotinib

## Abstract

Vamotinib (PF-114) is a 3rd -generation, ATP-competitive oral tyrosine kinase inhibitor (TKI) active against wild-type and mutated *BCR::ABL1* isoforms including *BCR::ABL1*^T315I^. We present final results of a phase-1 vamotinib dose-escalation study to identify maximum tolerated dose (MTD) and dose-limiting toxicity (DLT) followed by expansion cohorts. 51 subjects with chronic myeloid leukaemia (CML) failing ≥ 1 2nd generation TKI or with *BCR::ABL1*^T315I^ were enrolled. Subjects received vamotinib, 50–750 mg/d, continuously. Median exposure was 6 months (range, < 1–52 months). Median CML duration pre-study was 10 years (range, < 1–23 years). 27 subjects received ≥ 3 prior TKIs and 16 had *BCR::ABL1*^T315I^. The MTD was 600 mg with the Grade-3 psoriasis-like skin toxicity as the DLT. There were no vascular occlusive events nor deviations of ankle-brachial index. Complete haematologic response (CHR) was achieved in 14 of 30 subjects, major cytogenetic response (MCyR) in 14 of 44 subjects, complete cytogenetic response (CCyR) in 10 of 50 and major molecular response (MMR) in 7 of 51 subjects who did not have a CHR, MCyR, CCyR or MMR at enrollment. The best safety/efficacy dose was 300 mg with MCyR achieved in 6 of 7 subjects, CCyR in 5 of 9 and MMR in 4 of 9 subjects who did not have a MCyR, CCyR or MMR at enrollment. 5 of 16 subjects with *BCR::ABL1*^T315I^ responded including 3 achieving a CHR, 3, a MCyR, and 1,a CCyR. 2 of 5 subjects failing ponatinib achieved a CHR. Vamotinib dose for further phase-3 study is 300 mg/d.

**Supplementary Information:**

The online version contains supplementary material available at 10.1007/s00277-025-06239-8.

## Clinical trial registration

The trial is registered at *clinicaltrials.gov*. The registration number is NCT02885766, August 26, 2016.

## Introduction

Six tyrosine kinase inhibitors (TKIs) are approved to treat *BCR::ABL1* positive chronic myeloid leukemia (*BCR::ABL1* + CML) [1–6]. However, there remains a need for new TKIs because of resistance and intolerance [7–25]. Vamotinib (PF-114) mesylate is a potent, highly specific 3rd generation *BCR::ABL1*-inhibitor for oral use. Vamotinib is rationally designed to avoid inhibition of vascular endothelial growth factor receptor-2 (*VEGFR2*) and other off-target kinases [26–27]. Vamotinib is active against wild-type and mutant *BCR::ABL1* isoforms including *BCR::ABL1*^*T315I*^. In pre-clinical studies in animals there were few adverse events including no arterial occlusive events. We present the results of a phase-1 trial of vamotinib in subjects with *BCR::ABL1*-positive CML failing ≥ 2nd generation TKIs (2G-TKIs) or with *BCR::ABL1*^T315I^.

## Methods

### Study oversight

The study was designed by Fusion Pharma, LLC, in collaboration with the principal investigators from each clinical centre participating in the study and members of the sponsor’s Scientific Advisory Board. Data were collected, analyzed and interpreted by OCT (https://oct-clinicaltrials.com/) a clinical contract research organization (CRO). Principal investigators verified data accuracy and completeness at their site and integrity of the analyses and study conducted consistent with the study protocol.

### Subjects

Study subjects ≥ 18 years with *BCR::ABL1* positive CML in chronic (CP) or accelerated phase (AP) using the European LeukemiaNet 2013 guidelines [28]. Subjects, had to have failed ≥ 1 2nd generation TKIs or intolerance to ≥ 2 TKIs or have *BCR::ABL1*^T315I^. Other eligibility criteria included ECOG performance score 0–2; QTcF interval ≤ 470 milliseconds and resolution of all prior therapy-associated adverse event to ≤ Grade-1 according to NCI CTC AE v4 criteria [29].

### Study design

Open-label phase-1 study at 3 clinical centers with dose-escalation using a 3 + 3 scheme with a starting dose of 50 mg/d given continuously. A 28-days of therapy was defined as a cycle. Dose escalation for the subsequent cohort depended on the results of the safety assessment of subjects in the preceding cohort during the 28 days of therapy. Once an MTD was identified expanded cohorts of 10–15 subjects were recruited at doses of interest below the MTD. A modified accelerated dose-escalation regimen was used up to a dose of 400 mg. Dose could be increased for subjects in whom the dose was reduced because of an adverse drug reaction (ADR). Severity of adverse events was assessed using NCI CTC AE v4 [28].

The primary objective was to determine the MTD and DLT of vamotinib during the 1st cycle of therapy. Secondary objectives were to assess the safety and tolerability of vamotinib, determine pharmacokinetics and determine efficacy by response criteria according to 2013 ELN [29].

### Pharmaco-kinetics and -dynamics

Blood samples were obtained to evaluate pharmacokinetics and pharmacodynamics (Tables S1, S1.1, S1.2). Data on phosphorylated CrkL protein (pCrkL) concentration were evaluated in samples from 4 subjects.

### Efficacy

Haematological response evaluations were done on the first day of each cycle of therapy; molecular response on the first day of cycles 1, 2, 4, 7, 10, 13 and then every three months; cytogenetic response – at screening and on the first days of cycles 4, 7, 13 and every three months thereafter, provided that there was no major molecular response.

Cytogenetic response was assessed by differential staining of chromosomes and if uninformative, by fluorescent in situ hybridization (FISH). Mutations in *BCR::ABL1* were assessed by Sanger and next generation sequencing (NGS). Sanger sequencing was done at each center; NGS analyses were done in the laboratory of Prof. Susan Branford, University of Adelaide. Discordances were resolved as described in Table S2. Molecular response was assessed using the ratio of *BCR::ABL1* to *ABL1* measured according to the IS [9].

## Results

### Subjects and baseline co-variates

54 subjects were screened between July, 2016 and January, 2019, 51 of whom were enrolled. The starting dose of vamotinib was 50 mg/d (*n* = 3). Subsequent subjects were sequentially recruited into cohorts at doses of 100 mg (*n* = 3), 200 mg (*n* = 11), 400 mg (*n* = 12), 500 mg (*n* = 3), 600 mg (*n* = 6), 750 mg (*n* = 4), and 300 mg (*n* = 9). All subjects treated at 300 mg daily and 6 subjects at 400 mg were enrolled after the MTD was determined. The cut-off date of the analysis was July 30, 2019.

Baseline co-variates and therapy state of subjects as are displayed in Table 1. Mean follow-up is 27 w (range 0.1–31 w, IQR 65 w). 36 subjects previously received ≥ 3TKIs. 35 subjects had ≥ 1 *BCR::ABL1* kinase domain mutation the most common of which was *BCR::ABL1*^T315I^ in 16 (Table S2.1). All subjects discontinued from the study 1 of whom died later from blast transformation. 7 patients were remaining on the treatment when the study was terminated by the Sponsor’s decision (Table 1).

**Table 1:** Baseline co-variates and therapy state

	Cohort 1 50 mg (*N* = 3)	Cohort 2 100 mg (*N* = 3)	Cohort 3 200 mg (*N* = 11)	Cohort 4 400 mg (*N* = 12)	Cohort 5 500 mg (*N* = 3)	Cohort 6 600 mg (*N* = 6 )	Cohort 7 750 mg (*N* = 4)	Cohort 8 300 mg (*N* = 9)	All (*N* = 51)
**Time on therapy**, median (range)–days	308 (28–707)	394 (280–1592)	261 (45-1549)	162 (14–539)	54 (23–79)	102 (31-1002)	35 (13–364)	805 (77-1093)	199 (13-1592)
**Therapy state**									
Discontinued	3	3	11	12	3	6	4	9	51
Disease progression or need to change therapy in the opinion of the investigator	2	0	2	7	2	3	1	3	20
Adverse event	0	1	1	2	0	1	0	1	6
Intolerable adverse drug reaction in the opinion of the investigator	0	1	0	1	0	0	0	0	2
Withdrawal of informed consent	0	0	0	1	1	1	2	0	5
Participation in another clinical study or additional therapy	1	0	1	1	0	0	0	0	3
Lost to follow-up	0	0	1	0	0	0	0	1	2
Sponsor’s decision to terminate the study	0	1	2	0	0	1	0	3	7
Other	0	0	4	0	0	0	1	1	6
**ECOG performance-status score**									
0	3	2	8	9	2	5	2	9	40
1	0	1	3	3	0	1	2	0	10
2	0	0	0	0	1	0	0	0	1
**No. of previous TKIs**									
1	0	0	1	4	0	0	0	0	5
2	1	2	5	1	2	2	1	5	19
≥ 3	2	1	6	8	1	6	6	6	36
**Previous TKI**									
Imatinib	3	3	11	12	3	5	3	9	49
Nilotinib	2	2	4	7	2	5	4	6	32
Dasatinib	2	2	7	6	2	5	4	5	33
Bosutinib	1	0	4	1	0	2	3	3	14
Ponatinib	0	0	1	2	0	1	0	1	5
***BCR::ABL1*** **transcript**									
p 210	3	3	11	11	3	6	4	9	50
p 190 (e1a2)	0	0	0	1	0	0si	0	0	1

### Safety

There was no DLT in cohorts at doses < 400 mg/d. At a dose of 400 mg/d 1 subject developed a Grade-3 psoriasiform dermatitis (Table 2). There were no DLTs at doses of 500 mg/d and 600 mg/d. At a dose of 750 mg/d all 3 subjects treated developed skin DLTs, therefore 600 mg/d was defined as the MTD. Additional subjects were enrolled in the 200 mg/d, 300 mg/d, and 400 mg/d cohorts. Therapy duration was briefer at a dose of 400 md/d. Rates of dose-reductions and therapy-interruptions were higher for 400 mg/d compared with lower doses (Table 2).

**Table 2:** Subjects’ treatment and dose-limiting toxicities

Cohorts	Cohort 1 50 mg (*N* = 3)	Cohort 2 100 mg (*N* = 3)	Cohort 3 200 mg (*N* = 11)	Cohort 4 400 mg (*N* = 12)	Cohort 5 500 mg (*N* = 3)	Cohort 6 600 mg (*N* = 6)	Cohort 7 750 mg (*N* = 4)	Cohort 8 300 mg (*N* = 9)	All subjects, (*N* = 51)
Time on treatment, median (range)– days	308 (28–707)	394 (280–1592)	261 (45-1549)	162 (14–539)	54 (23–79)	102 (31-1002)	35 (13–364)	805 (77-1093)	199 (13-1592)
DLTs	0	0	0	1	0	0	3	0	4
Subjects with dose delay, n	1	2	6	6	1	4	3	4	27
Subjects with dose reduction, n	1	1	3	4	1	2	3	0	15

The most common non-hematological adverse event was psoriasiform dermatitis in 19 subjects 4 of which were Grade-3/-4 (Table 3). There was diarrhea in 14 subjects, all < Grade-3. Dry skin and rash occurred in 8 subjects 6 subjects had a Grade-3/-4 toxic skin rash. 6 subjects had serious adverse events (Table S3).

Haematological adverse events were common but usually Grade-1/-2 (Table 3). No cardio-vascular or arterial occlusive adverse events were reported at any dose, including no cases of occlusion of large peripheral blood vessels and no clinically significant deviations of the ankle-brachial index from its normal range 1.00-1.29 [30]. The 3 observed cases of drug-related cardiac disorders were Grade-2 atrial fibrillation (400 mg), Grade-1 extrasystoles (600 mg) and Grade-1 pericarditis (400 mg).

**Table 3:** Most common adverse events

Adverse Events	All subjects (*N* = 51)		Advanced phase (*N* = 5)
	All grades	Grade 3–4^*^	Grade 3–4^*^
	*Number of subjects (percent)*		
**Skin and subcutaneous tissue disorders**	**32 (63)**	**11 (22)**	**1 (20)**
Dermatitis psoriasiform	19 (37)	4 (8)	
Toxic skin eruption	9 (18)	7 (14)	1 (20)
Dry skin	9 (18)		
Pruritus	5 (10)	1 (2)	
Erythema	4 (8)		
Rash papular	2 (4)		
**Gastrointestinal disorders**	**17 (33)**		
Diarrhoea	14 (28)		
Nausea	6 (12)		
Abdominal pain upper	4 (8)		
Abdominal pain	2 (4)		
Anal fissure	2 (4)		
Dyspepsia	2 (4)		
**Blood and lymphatic system disorders**	**12 (24)**	**7 (14)**	**2 (40)**
Thrombocytopenia	8 (16)	4 (8)	1 (20)
Neutropenia	5 (10)	3 (6)	2 (40)
Anaemia	2 (4)	1 (2)	
Leukopenia	1 (2)	1 (2)	
**Investigations**	**7 (14)**	**1 (2)**	
Blood cholesterol increased	3 (6)		
Cardiac disorders	3 (6)		
Eye disorders	3 (6)		
Alanine aminotransferase increased	2 (4)		
Low density lipoprotein increased	2 (4)		
Transaminases increased	1 (2)	1 (2)	
**Hepato-biliary disorders**	**1 (2)**	**1 (2)**	
Hepatitis toxic	1 (2)	1 (2)	

^*^Dermatitis psoriasiform – 2 subjects on 400 mg; 2 subjects on 600 mg; Toxic skin eruption – 1 subject on 300 mg; 1 subject on 400 mg; 2 subjects on 500 mg; 1 subject on 600 mg; 2 subjects on 750 mg; Pruritus – 1 subject on 600 mg; Hepatitis toxic – 1 subject on 400 mg; Thrombocytopenia – 1 subject on 200 mg; 1 subject on 500 mg; 1 subject on 600 mg; 1 subject on 750 mg; Neutropenia – 2 subjects on 400 mg; 1 subject on 500 mg; Anaemia – 1 subject on 200 mg; Leukopenia – 1 subject on 400 mg

### Rash

Rash was the most common non-haematological adverse event and were seen in 32 subjects (Table 4). Grade-3 rash occurred in 10 subjects at doses ≥ 400 mg/d. Rash was the reason for dose-reduction (*n* = 9) and study withdrawal (*n* = 6). No rash was coded as a serious adverse events. The most common rash was psoriasiform with erythema, lymphoplasmacytic infiltration of derma and desquamation of well-defined foci. Rashes were often in sites of friction and stretching and painless but occasionally associated with paresthesia. Other types of rashes included dryness and redness mainly in the setting of development or regression of psoriasiform rashes. Toxicity resolved within 1–2 weeks after discontinuation of the drug. Lipoic acid (tablets), vitamin E, and topical drugs including tacrolimus 0.1, betamethasone 0.05, calcipotriol, hydrocortisone 0.5 eye ointment, urea 10, thermal water were given for skin lesions. Skin lesions were resolved after 4–6 months.

**Table 4:** Skin toxicity

	Severity	Cohort 1 50 mg	Cohort 2 100 mg	Cohort 3 200 mg	Cohort 4 400 mg	Cohort 5 500 mg	Cohort 6 600 mg	Cohort 7 750 mg	Cohort 8 300 mg	Overall
		*N* = 3	*N* = 3	*N* = 11	*N* = 12	*N* = 3	*N* = 6	*N* = 4	*N* = 9	*N* = 51
Psoriasiform dermatitis, n	Grade 3	0	0	1	2	0	1	0	0	4
	Total	1	3	4	8	0	1	0	2	19
Dry skin, n	Grade 3	0	0	0	0	0	0	0	0	0
	Total	0	1	1	4	1	1	0	1	9
Erythema, n	Grade 3	0	0	0	0	0	0	0	0	0
	Total	0	0	0	2	1	1	0	0	4
Pruritus, n	Grade 3	0	0	0	0	0	1	0	0	1
	Total	0	0	0	3	0	2	0	0	5
Toxic skin eruption, n	Grade 3	0	0	1	1	1	1	3	0	7
	Total	0	0	1	1	1	3	3	0	9
Rash papular, n	Grade 3	0	0	0	0	0	0	0	0	0
	Total	1	0	0	1	0	0	0	0	2

### Pharmacokinetics

**Fig. 1:**
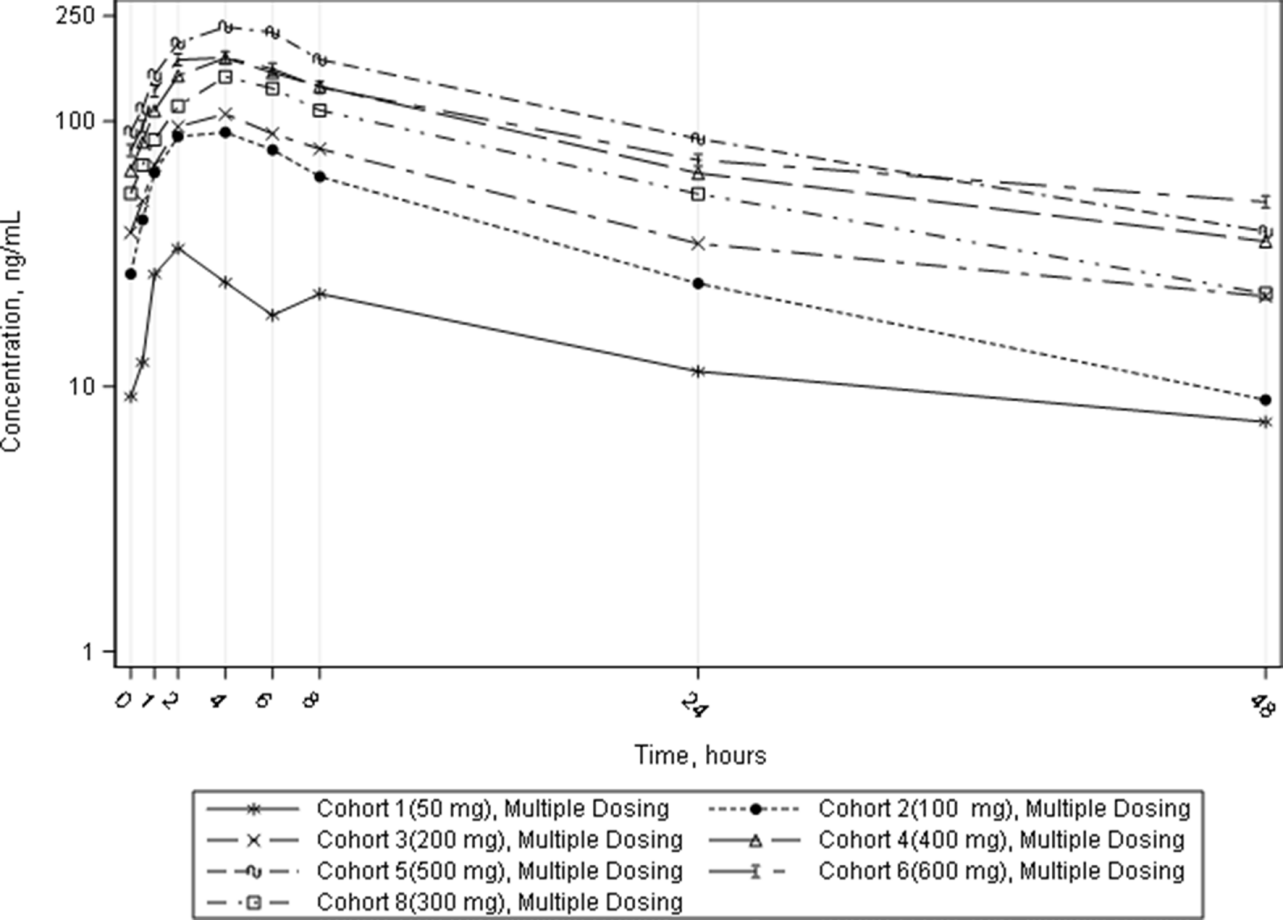
Vamotinib concentrations after multiple-dose administration (Days 1–2 of the cycle 2 of therapy) of vamotinib mesylate for all subjects by dosage cohorts (population for PK evaluation)

Pharmacokinetic measurements were done on days 1 and 29–30 of treatment. The time to reach C_max_ was about 4 h, the half-life of 13.5 h, which confirmed the validity of the once a day regimen. Steady-state was reached after 8 days of dosing, Fig. 1. The concentration-time profiles suggest linear or near-linear pharmacokinetics of vamotinib over the entire dose range studied (50–750 mg). Dose proportionality assessment showed a less than dose-dependent increase in AUC and C_max_ of vamotinib (Table S4). Starting from a dose of 200 mg, the concentration of vamotinib in the blood during the day exceeds 75 nM, corresponding to the IC50 of cytotoxicity for BaF3 cells expressing *BCR::ABL1*^*T315I*^ [26].

Analysis of changes in blood pCrkL done for subjects with leukocytosis on days 1, 8 and 29 of therapy but was uninformative because of poor quality of samples.

### Efficacy

Efficacy and best responses to treatment are summarized in Table 5 and Table S5 correspondingly. 30 subjects did not have a complete hematological response (CHR) at baseline 14 of whom achieved a CHR (Table 5). Median time to achieving a CHR was 3.8 months, (range, 1–4 months). Of the 44 and 50 subjects who did not have a major cytogenetic response (MCyR) and a complete cytogenetic response (CCyR) at the start of therapy, respectively, 14 (32%) achieved MCyR and 10 (20%) achieved CCyR during therapy. The median time to achievement of MCyR and CCyR were 4.5 months (range 1.9–19.4) and 5.6 months (range 2.8–15), respectively. None of the 51 subjects had a major molecular response (MMR) at study entry, and 7 (13.7%) subjects achieved MMR during therapy. One subject on a dose of 300 mg achieved a deep molecular response of MR4.5. It is worth mentioning that 4 of 7 subjects achieving MMR had intolerance to prior TKI therapy before enrollment to the study (Table S5). The median time to MMR was 5,6 months (range 2.8–22.2), and no subject has lost after 24 months. One subject, treated at a dose of 300 mg, achieved a MR4.5. In general, the higher the level of response to therapy was achieved the more sustainable it was: 8 of 14 CHRs were subsequently lost, whereas none of 7 MMRs were lost. Most molecular and cytogenetic responses were observed at doses of 300 and 200 mg. The lower number of responses at doses of 400 mg and above may be explained by the fact that the resulting (skin) toxicity prevented long-term treatment of subjects at these doses.

**Table 5:** Efficacy

	Cohort 1 50 mg	Cohort 2 100 mg	Cohort 3 200 mg	Cohort 4 400 mg	Cohort 5 500 mg	Cohort 6 600 mg	Cohort 7 750 mg	Cohort 8 300 mg	Total
	*N* = 3	*N* = 3	*N* = 11	*N* = 12	*N* = 3	*N* = 6	*N* = 4	*N* = 9	*N* = 51
Absence of CHR at baseline – no. (%)	2 (67)	0	5 (46)	7 (58)	2 (67)	6 (100)	4 (100)	4 (44)	30 (59)
CHR achieved at treatment – no. (%)	1 (50)	0	2 (40)	4 (57)	0	2 (33)	1 (25)	4 (100)	14 (47)
CHR by the 3 months – no. (%)	0	0	2 (40)	2 (29)	0	2 (33)	1 (25)	4 (100)	11 (37)
CHR by the 6th months – no. (%)	0	0	2 (40)	2 (29)	0	2 (33)	1 (25)	4 (100)	11 (37)
CHR by the 12th months – no. (%)	1 (50)	0	2 (40)	4 (57)	0	2 (33)	1 (25)	4 (100)	14 (47)
Subjects with loss of CHR – no. (%)	0	0	2 (100)	4 (100)	0	0	0	2 (50)	8 (57)
Absence of MCyR at baseline – no. (%)	3 (100)	2 (67)	8 (73)	11 (92)	3 (100)	6 (100)	4 (100)	7 (78)	44 (86)
MCyR achieved at treatment – no. (%)	0	2 (67)	4 (50)	2 (19)	0	0	0	6 (86)	14 (32)
MCyR by 6 months – no. (%)	0	1 (33)	3 (38)	2 (19)	0	0	0	4 (57)	10 (23)
MCyR by 12 months – no. (%)	0	1 (33)	3 (38)	2 (19)	0	0	0	5 (83)	11 (25)
Subjects with loss of MCyR – no. (%)	0	0	1 (25)	0	0	0	0	1 (17)	2 (14)
Absence of CCyR at baseline – no. (%)	3 (100)	3 (100)	10 (91)	12 (100)	3 (100)	6 (100)	4 (100)	9 (100)	50 (98)
CCyR achieved at treatment – no. (%)	0	1 (33)	3 (30)	1 (8)	0	0	0	5 (56)	10 (20)
CCyR by 6 months – no. (%)	0	0	2 (20)	1 (8)	0	0	0	3 (33)	6 (12)
CCyR by 12 months – no. (%)	0	0	2 (20)	1 (8)	0	0	0	4 (44)	7 (14)
Subjects with loss of CCyR – no. (%)	0	0	1 (33)	0	0	0	0	1 (20)	2 (20)
Absence of MMR at baseline – no. (%)	3 (100)	3 (100)	11 (100)	12 (100)	3 (100)	6 (100)	4 (100)	9 (100)	51 (100)
MMR achieved at treatment – no. (%)	0	1 (33)	2 (18)	0	0	0	0	4 (44)	7 (14)
MMR by 6 months – no. (%)	0	0	1 (9)	0	0	0	0	3 (33)	4 (8)
MMR by 12 months – no. (%)	0	0	1 (9)	0	0	0	0	3 (33)	4 (8)
MMR by 24 months – no. (%)	0	1 (33)	2 (18)	0	0	0	0	4 (44)	7 (14)
Subjects with loss of MMR – no. (%)	0	0	0	0	0	0	0	0	0

Of the 16 subjects with *BCR::ABL1*^T315I^, 12 did not have CHR at study entry; 3 of them achieved CHR during vamotinib therapy. Of the 15 subjects without MCyR and 16 without CCyR, 3 and 1 subjects achieved responses, respectively (Table 6). Responses to vamotinib in subjects with *BCR::ABL1*^T315I^ were transient, lasting 2.8 months (MCyR) and 2.8 months (CCyR). Most subjects with *BCR::ABL1*^T315I^ were treated at doses of 400 mg and above, which were characterized by premature withdrawal from the study due to toxicity. No subject with *BCR::ABL1*^T315I^ was included in the 300 mg dose cohort, which performed optimally in terms of efficacy and safety. Five subjects had received prior therapy ponatinib. None had CHR at study entry and 2 achieved CHR; however, none achieved MCyR or CCyR (Table 6). Five subjects with advanced phase CML (4 in AP and 1 in blast phase, BP) were treated. 2 achieved a CHR but none achieved MCyR or CCyR. 1 subject continued on therapy (Table 6).

A subject with myeloid variant M0 blast crisis was treated with vamotinib due to the lack of other available at that time therapies [31–32]. BP was identified on enrollment and accompanied by i(17) and *BCR::ABL1*^T315I^. A CHR was achieved after 5 weeks of vamotinib lasting for 18 months with no cytogenetic response. At 1 year there was extra-medullary relapse and the CHR was lost and vamotinib discontinued.

**Table 6:** Subjects with the *BCR::ABL1*^T315I^, resistance/intolerance to ponatinib or in accelerated phase

Variable	Subjects with BCR::ABL1^T315I^ (*N* = 16)	Subjects after ponatinib (*N* = 5)	Advanced phase CML (*N* = 5)
Median follow-up (range), days	78 (17–987)	53 (8-266)	150 (15–539)
CHR no./total no.	3/12^*^	2/5	2/5
MCyR no./total no.	3/15^**^	0/5	0/5
CCyR no./total no.	1/16	0/5^*^	0/5
MMR no./total no.	0/16	0/5^*^	0/5

^*^ 4 subjects had a CHR at enrollment

^**^ 1subject had a MCyR at enrollment

## Discussion

Vamotinib is safe and reasonably effective in people with CML resistant and/or intolerance to 1st and 2nd generation TKIs, ponatinib and in those with *BCR::ABL1*^T315I^. The MTD is 600 mg with rash the dose-limiting and most common DLT and adverse event. Rash was seen in pre-clinical toxicology studies in rats and dogs [26]. PDGFRa seems the most likely off-target kinase causing skin toxicity of vamotinib, as follows from the kinase inhibition profiles of vamotinib, ponatinib and dasatinib [26]. Ponatinib and vamotinib, known to cause skin toxicity, potently inhibited PDGFRa, unlike dasatinib, for which skin toxicity is not characteristic. PDGFRa was also spotted among the off-target kinases of another *BCR::ABL1* kinase inhibitor olverembatinib [33], which is currently under intensive clinical evaluation [34–35] and for which skin pigmentation was the most common non-hematologic adverse reaction [34]. Interestingly, vamotinib showed no arterial-occlusive events despite the close structural similarity with ponatinib [23–24]. Comparison of kinase inhibition profiles of ponatinib and vamotinib [26] may suggest that inhibition of EPHA6, EPHA7, TAK1, TIE2, VEGFR2, ZAK kinases by ponatinib but not vamotinib may contribute to cardiovascular toxicity of the former. No cardio-vascular findings were observed in pre-clinical toxicology studies of vamotinib as well [26]. Recent clinical studies of olverembatinib, also a structural homologue of ponatinib, revealed some drug related cardiovascular adverse events, including vascular occlusions, however potentially less common compared to ponatinib [34]. Olverembatinib is a potent inhibitor of discussed above kinases TAK1, TIE2 and ZAK, but not EPHA6, EPHA7 and VEGFR2 [33], which puts it somewhere between ponatinib and vamotinib.

The study has its limitations due to the relatively small sample size, heterogeneous pretreatment status of the enrolled patients, and the lack of reliable pharmacodynamic readouts. Despite the small statistics, promising efficacy of vamotinib was observed in the 300 mg cohort where 4 of 9 patients achieved MMR, Some patients with *BCR::ABL1*^*T315I*^ achieved MCyR and CCyR. However no patients with *BCR::ABL1*^*T315I*^ were enrolled in 300 mg cohort in this study, so more data are needed to characterize the efficacy of vamotinib 300 mg in subjects with *BCR::ABL1*^*T315I*^. Previous data on ponatinib [21], asciminib [6] and olverembatinib [34–35] suggest comparable efficacy of these drugs in subjects with or without *BCR::ABL1*^*T315I*^.

In conclusion, vamotinib is safe and effective in some subjects failing ≥ 2 TKIs, ponatinib and with *BCR::ABL1*^T315I^. The dose selected for further study is 300 mg/d. Vamotinib is currently being evaluated in a phase-3 study versus high-dose imatinib in subjects resistance to standard-dose imatinib without *BCR::ABL1* mutations known to confer resistance to imatinib [36].

## Electronic supplementary material

Below is the link to the electronic supplementary material.

Supplementary Material 1

## Data Availability

No datasets were generated or analysed during the current study.
