# Supplementary material for: Phase-1 study of vamotinib (PF-114), a 3rd generation BCR::ABL1 tyrosine kinase-inhibitor, in chronic myeloid leukaemia
Source: Ann Hematol. 2025 Apr 29;104(5):2707–15. doi: 10.1007/s00277-025-06239-8 (PMC12141164; doi:10.1007/s00277-025-06239-8)
Supplement: Supplementary file 1 — Supplementary Material 1 [file 277_2025_6239_MOESM1_ESM.docx]

# Supplementary Information

## **Table S1** Pharmacokinetic and pharmacodynamic sampling

| Procedure | Screening | | Cycle 1 | | | | | Cycle 2 | | | |
| --- | --- | --- | --- | --- | --- | --- | --- | --- | --- | --- | --- |
|  | Day | | Day of Cycle 1 | | | | | Day of Cycle 2 | | | |
|  | < -21 | -7 to 1 | 1 | 2 | 8* | 15* | 22* |  |  |  |  |
| **Medication with PF-114 mesylate** |  | | Every day, once daily | | | | | Every day once daily except for Day 2 | | | |
| **Pharmacodynamic response sampling**  The samples for pharmacodynamic assessment (measuring of pCrkL protein level) must be taken only from patients who are not in complete hematologic response at enrollment into the study. The time points of sampling for pharmacodynamic assessment are provided in the Table S1.1. |  |  | X | X | X |  |  | X |  |  |  |
| **Pharmacokinetic sampling**  The time points of pharmacokinetic sampling are provided in the Table S1.2. The 24-hour sample must be taken before the second dose of the study drug on Day 2 Cycle 1. |  |  | X | X | X |  |  | X | X | X |  |

## ^*^Procedures and samplings may be performed within ± 3 days from the scheduled date.

##

## **Table S1.1** Time points for pharmacokinetic sampling

| Day | Time points for sampling (in hours from administration of the study drug) |
| --- | --- |
| Cycle 1, Day 1 | Pre-dose*  0,5 ± 5 minutes  1 ± 5 minutes  2 ± 15 minutes  4 ± 15 minutes  6 ± 15 minutes  8 ± 15 minutes |
| Cycle 1, Day 2 | Pre-dose (after 24 ± 1 hours post-dose)* |
| Cycle 1, Day 8 | Pre-dose * |
| Cycle 2, Day 1 | Pre-dose *  0,5 ± 5 minutes  1 ± 5 minutes  2 ± 15 minutes  4 ± 15 minutes  6 ± 15 minutes  8 ± 15 minutes |
| Cycle 2, Day 2 | 24 ± 1 hours post dose **(the study drug is not taken)** |
| Cycle 2, Day 3 | Pre-dose (after 48 ± 1 hours post-dose) |

^*^ Pharmacodynamic sampling is performed as well

## **Table S1.2** Time points for pharmacodynamic sampling

| Day | Time points for sampling (in hours) |
| --- | --- |
| Cycle 1, Day 1 | Pre-dose* |
| Cycle 1, Day 2 | Pre-dose (after 24 hours post-dose)* |
| Cycle 1, Day 8 | Pre-dose * |
| Cycle 2, Day 1 | Pre-dose |

* Pharmacokinetic sampling is performed as well

**Table S2** Consensus approach to assign patient’s mutation in *BCR::ABL1* when discordances between Sanger and NGS outcomes are revealed.

| **Outcome of Sanger sequencing** | **Outcome of NGS Sequencing** | **Consensus mutation** |
| --- | --- | --- |
| Mutation 1 | Mutation 2 | Mutation 2 |
| Mutation 1 | Mutation is absent / no data | Mutation 1 |
| Mutation is absent / no data | Mutation 2 | Mutation 2 |
| No data | Mutation is absent | Mutation is absent |
| Mutation is absent | No data | Mutation is absent |
| Mutation is absent | Mutation is absent | Mutation is absent |
| No data | No data | Unknown |

**Table S2.1** Distribution of transcript types and mutations by cohorts

|  | Cohort 1 (50 mg) (N=3) | Cohort 2 (100 mg) (N=3) | | Cohort 3 (200 mg) (N=11) | | Cohort 4 (400 mg) (N=12) | | Cohort 5 (500 mg) (N=3) | | Cohort 6 (600 mg) (N=6) | | Cohort 7 (750 mg) (N=4) | | Cohort 8 (300 mg) (N=9) | | Overall (N=51) | |
| --- | --- | --- | --- | --- | --- | --- | --- | --- | --- | --- | --- | --- | --- | --- | --- | --- | --- |
| *BCR::ABL1* mutations, consensus |  | |  | |  | |  | |  | |  | |  | |  | |  |
| Absent | 1 (33.3) | 2 (66.7) | | 9 (81.8) | | 1 (8.3) | | 0 | | 1 (16.7) | | 1 (25.0) | | 6 (66.7) | | 21 (41.2) | |
| D276G | 0 | 0 | | 0 | | 1 (8.3) | | 1 (33.3) | | 0 | | 0 | | 0 | | 2 (3.9) | |
| E282Q | 1 (33.3) | 0 | | 0 | | 0 | | 0 | | 0 | | 0 | | 0 | | 1 (2.0) | |
| E355A | 0 | 0 | | 0 | | 1 (8.3) | | 0 | | 0 | | 0 | | 0 | | 1 (2.0) | |
| F311L | 0 | 0 | | 0 | | 0 | | 0 | | 0 | | 0 | | 1 (11.1) | | 1 (2.0) | |
| F359V | 0 | 0 | | 0 | | 2 (16.7) | | 0 | | 0 | | 0 | | 1 (11.1) | | 3 (5.9) | |
| G250E | 0 | 0 | | 0 | | 1 (8.3) | | 0 | | 0 | | 0 | | 0 | | 1 (2.0) | |
| L248V | 0 | 0 | | 0 | | 0 | | 0 | | 1 (16.7) | | 0 | | 0 | | 1 (2.0) | |
| T315I | 1 (33.3) | 1 (33.3) | | 1 (9.1) | | 4 (33.3) | | 1 (33.3) | | 2 (33.3) | | 1 (25.0) | | 0 | | 11 (21.6) | |
| T315I/ F317L | 0 | 0 | | 1 (9.1) | | 0 | | 1 (33.3) | | 0 | | 0 | | 0 | | 2 (3.9) | |
| T315I/ F359V | 0 | 0 | | 0 | | 0 | | 0 | | 1 (16.7) | | 1 (25.0) | | 0 | | 2 (3.9) | |
| T315I/ G250E/ L248V | 0 | 0 | | 0 | | 0 | | 0 | | 0 | | 1 (25.0) | | 0 | | 1 (2.0) | |
| V299L | 0 | 0 | | 0 | | 1 (8.3) | | 0 | | 1 (16.7) | | 0 | | 0 | | 2 (3.9) | |
| Y215_Y342DEL | 0 | 0 | | 0 | | 1 (8.3) | | 0 | | 0 | | 0 | | 0 | | 1 (2.0) | |
|  | | | | | | | | | | | | | | | | | |

## **Table S3** Serious adverse events

| Serious adverse event | | Relation to study drug | Cohort 1 (50 mg) (N=3) n (%) / E | Cohort 2 (100 mg) (N=3) n (%) / E | Cohort 3 (200 mg) (N=11) n (%) / E | Cohort 4 (400 mg) (N=12) n (%) / E | Cohort 5 (500 mg) (N=3) n (%) / E | Cohort 6 (600 mg) (N=6) n (%) / E | Cohort 7 (750 mg) (N=4) n (%) / E | Cohort 8 (300 mg) (N=9) n (%) / E | Overall (N=51) |
| --- | --- | --- | --- | --- | --- | --- | --- | --- | --- | --- | --- |
| **Overall** | |  | **0** | **0** | **0** | **1 (8.3)/1** | **0** | **2 (33.3)/2** | **1 (25.0)/1** | **3 (33.3)/3** | **7 (13.7)/7** |
|  |  | | | | | | | | | | |
| **Cardiac disorders** | |  | **0** | **0** | **0** | **1 (8.3)/1** | **0** | **0** | **0** | **0** | **1 (2.0)/1** |
| Atrial fibrillation | | Yes | 0 | 0 | 0 | 1 (8.3)/1 | 0 | 0 | 0 | 0 | 1 (2.0)/1 |
|  |  | | | | | | | | | | |
| **General disorders and administration site conditions** | |  | **0** | **0** | **0** | **0** | **0** | **0** | **1 (25.0)/1** | **0** | **1 (2.0)/1** |
| Disease progression | | No | 0 | 0 | 0 | 0 | 0 | 0 | 1 (25.0)/1 | 0 | 1 (2.0)/1 |
|  |  | | | | | | | | | | |
| **Infections and infestations** | |  | **0** | **0** | **0** | **0** | **0** | **2 (33.3)/2** | **0** | **3 (33.3)/3** | **5 (9.8)/5** |
| Acute sinusitis | | No | 0 | 0 | 0 | 0 | 0 | 1 (16.7)/1 | 0 | 0 | 1 (2.0)/1 |
| Appendicitis | | No | 0 | 0 | 0 | 0 | 0 | 0 | 0 | 1 (11.1)/1 | 1 (2.0)/1 |
| Peritonitis | | No | 0 | 0 | 0 | 0 | 0 | 0 | 0 | 1 (11.1)/1 | 1 (2.0)/1 |
| Pneumonia | | No | 0 | 0 | 0 | 0 | 0 | 0 | 0 | 1 (11.1)/1 | 1 (2.0)/1 |
| Upper respiratory tract infection | | No | 0 | 0 | 0 | 0 | 0 | 1 (16.7)/1 | 0 | 0 | 1 (2.0)/1 |
|  |  | | | | | | | | | | |

Table displays number of patients (n), percentage (based on N) and number of events (E) in category by dose cohort.

##

## **Table S4** Pharmacokinetic parameters of vamotinib mesylate after single- and multiple-dose administration by dosage cohorts

|  | Cohort 1: 50 mg | Cohort 2: 100 mg | Cohort 3: 200 mg | Cohort 4: 400 mg | Cohort 5: 500 mg | Cohort 6: 600 mg | Cohort 7: 750 mg | Cohort 8: 300 mg |
| --- | --- | --- | --- | --- | --- | --- | --- | --- |
| **Single-dose administration, AUC0-t (ng*h/ml)** | | | | | | | | |
| n | 3 | 3 | 10 | 11 | 2 | 6 | 4 | 9 |
| Mean | 252,3 | 1072,7 | 1064,9 | 1742,8 | 1497,0 | 3018,8 | 3292,0 | 1626,0 |
| CV, % | 35,3 | 43,1 | 55,7 | 54,7 | 9,8 | 39,6 | 38,1 | 38,0 |
| **Single-dose administration, Cmax (ng/ml)** | | | | | | | | |
| n | 3 | 3 | 10 | 11 | 2 | 6 | 4 | 9 |
| Mean | 25,30 | 89,73 | 82,14 | 119,50 | 115,50 | 211,67 | 224,75 | 125,12 |
| CV, % | 38,0 | 38,9 | 56,4 | 52,5 | 8,0 | 41,8 | 36,0 | 26,4 |
| **Single-dose administration, T1/2 (h)** | | | | | | | | |
| n | 3 | 2 | 9 | 7 | 1 | 4 | 3 | 9 |
| Median | 11,300 | 10,190 | 10,500 | 12,100 | 11,500 | 10,740 | 10,600 | 10,400 |
| Minimum | 10,30 | 9,08 | 8,63 | 9,61 | 11,50 | 8,93 | 8,71 | 7,36 |
| Maximum | 13,20 | 11,30 | 15,50 | 19,60 | 11,50 | 17,70 | 12,80 | 11,90 |
| **Single-dose administration, Tmax (h)** | | | | | | | | |
| n | 3 | 3 | 10 | 11 | 2 | 6 | 4 | 9 |
| Mean | 2,667 | 4,667 | 3,465 | 4,722 | 3,960 | 4,653 | 4,505 | 3,579 |
| CV, % | 43,3 | 24,2 | 27,8 | 38,6 | 72,9 | 22,4 | 22,1 | 24,8 |
| **Multiple-dose administration, AUC0-24 (ng*h/ml)** | | | | | | | | |
| n | 2 | 3 | 9 | 8 | 2 | 4 | 0 | 8 |
| Mean | 445,0 | 1255,7 | 1580,6 | 2711,1 | 3491,5 | 2800,8 |  | 2264,5 |
| CV, % | 44,8 | 11,4 | 48,1 | 29,8 | 57,3 | 59,3 |  | 30,6 |
| **Multiple-dose administration, Cmax (ng/ml)** | | | | | | | | |
| n | 2 | 3 | 9 | 8 | 2 | 4 | 0 | 9 |
| Mean | 33,30 | 94,03 | 109,14 | 177,38 | 227,50 | 179,25 |  | 148,01 |
| CV, % | 22,9 | 7,2 | 58,8 | 27,7 | 44,4 | 52,1 |  | 32,2 |
| **Multiple-dose administration, Ctrough (ng/ml)** | | | | | | | | |
| n | 2 | 3 | 9 | 8 | 2 | 4 | 0 | 8 |
| Mean | 7,35 | 8,90 | 21,92 | 35,34 | 38,40 | 49,78 |  | 22,45 |
| CV, % | 72,2 | 36,8 | 78,5 | 40,0 | 53,8 | 136,9 |  | 53,8 |
| **Multiple-dose administration, T1/2 0-24 (h)** | | | | | | | | |
| n | 1 | 3 | 9 | 7 | 2 | 3 | 0 | 7 |
| Median | 16,100 | 12,100 | 11,800 | 14,700 | 13,650 | 11,800 |  | 13,000 |
| Minimum | 16,10 | 9,25 | 10,30 | 11,10 | 13,30 | 9,85 |  | 9,77 |
| Maximum | 16,10 | 12,20 | 20,00 | 18,60 | 14,00 | 23,20 |  | 22,90 |
| **Multiple-dose administration, Tmax (h)** | | | | | | | | |
| n | 2 | 3 | 9 | 8 | 2 | 4 | 0 | 9 |
| Mean | 1,900 | 3,360 | 3,364 | 3,995 | 4,040 | 3,625 |  | 4,523 |
| CV, % | 5,2 | 35,1 | 31,9 | 26,8 | 1,4 | 53,3 |  | 19,7 |

## **Table S5** Complete list of patient responses with characteristics of previous therapy and consensus mutation

| **ID** | **Dose cohort, mg** | **Duration of CML, years** | **Prior CML therapy with a TKI**  **(R – rsistance, I - intolerance)** | **Abl consensus mutation at enrollment** | **Abl consensus mutation at end of treatment** | **Best response achieved on PF-114** |
| --- | --- | --- | --- | --- | --- | --- |
| 101 | 50 | 18 | bosutinib R  dasatinib R  imatinib R | E282Q | **Absent** | CHR |
| 102 | 50 | 6 | imatinib I, R  nilotinib R | T315I | T315I |  |
| 103 | 50 | 13 | imatinib I, R  nilotinib I, R  dasatinib R | Absent | Absent |  |
| 104 | 100 | 9 | imatinib R  nilotinib R  dasatinib I, R | T315I | T315I | MCyR |
| 105 | 100 | 5 | imatinib R  nilotinib I, R | Absent | Absent |  |
| 106 | 100 | 15 | imatinib R  dasatinib I | Absent | Absent | MMR |
| 107 | 125 | 23 | imatinib I, R  dasatinib I | Absent | Absent | MR 4.5 |
| 108 | 200 | 14 | imatinib R  bosutinib R  dasatinib R  nilotinib I | Absent | Unknown | CHR |
| 109 | 200 | 8 | imatinib R | T315I | T315I | MCyR |
| 110 | 400 | 4 | imatinib R  nilotinib R | F359V | F359V  **F359C** |  |
| 111 | 400 | 5 | imatinib R | D276G | **T315I** | CHR |
| 112 | 400 | 17 | imatinib I | Absent | **Y253H** | CHR |
| 113 | 400 | 9 | imatinib I  nilotinib R  dasatinib R | T315I | **Absent** | CCyR |
| 114 | 400 | 14 | imatinib R  nilotinib R  dasatinib R | G250E | G250E  **G749A** |  |
| 115 | 400 | 4 | imatinib R  nilotinib R  ponatinib R | T315I | Unknown |  |
| 116 | 400 | 11 | imatinib I, R | Y215_Y342DEL | **Absent** | CHR |
| 117 | 400 | 10 | imatinib R | T315I | T315I  **F359V** |  |
| 118 | 400 | 5 | imatinib I  nilotinib R  dasatinib R  ponatinib I | F359V | F359V |  |
| 119 | 600 | 3 | imatinib R  dasatinib R | T315I | T315I |  |
| 121 | 200 | 3 | imatinib I, R  dasatinib R  ponatinib I | Absent | Unknown |  |
| 122 | 300 | 20 | imatinib R  nilotinib R | Absent | Absent | MMR |
| 123 | 300 | 15 | imatinib R  nilotinib R  ponatinib R | Unknown | Unknown | CHR |
| 124 | 200 | 12 | imatinib I  bosutinib R | Absent | T315I |  |
| 125 | 200 | 4 | imatinib R  nilotinib I  dasatinib R | T315I  F317L | **Absent** |  |
| 126 | 200 | 14 | imatinib R  dasatinib I  bosutinib I | Absent | Unknown | CCyR |
| 201 | 400 | 18 | imatinib R  nilotinib R  dasatinib I | E355A | **E355G** | MCyR |
| 202 | 400 | 15 | imatinib R  dasatinib R  bosutinib R | V299L | V299L | CHR |
| 203 | 500 | 17 | imatinib R  dasatinib R | T315I  **F317L** | T315I |  |
| 204 | 500 | 17 | imatinib R  nilotinib R  dasatinib R | T315I | T315I |  |
| 205 | 500 | 14 | imatinib R  nilotinib R | D276G | **Absent** |  |
| 206 | 400 | 12 | nilotinib I  imatinib I, R  dasatinib R | T315I | T315I |  |
| 207 | 600 | 17 | imatinib R  dasatinib R  nilotinib R | L248V | L248V |  |
| 209 | 600 | 3 | imatinib R  nilotinib R | T315I  **F359V** | T315I |  |
| 210 | 750 | 5 | nilotinib R  dasatinib R | T315I  **F359V** | T315I |  |
| 211 | 600 | 7 | nilotinib I, R  dasatinib R  bosutinib R  ponatinib R | T315I | **Absent** | CHR |
| 212 | 200 | 2.5 | imatinib R  nilotinib R  dasatinib R | Absent | Unknown |  |
| 301 | 300 | 14 | imatinib R  nilotinib R  bosutinib R  dasatinib R | Absent | Unknown | MR 4 |
| 302 | 300 | 11 | imatinib R  nilotinib R  bosutinib R  dasatinib R | Absent | Unknown | CHR |
| 303 | 300 | 17 | imatinib R  dasatinib I, R | Absent | Absent | CCyR |
| 304 | 750 | 9 | imatinib R  nilotinib R  bosutinib R  dasatinib I, R | T315I | T315I |  |
| 305 | 300 | 6 | imatinib R  nilotinib R  bosutinib R | Absent | **T315I** | CCyR |
| 306 | 750 | 24 | imatinib R  nilotinib I  dasatinib R  bosutinib R | Absent | Unknown | CHR |
| 307 | 750 | 5 | imatinib R  nilotinib R  bosutinib R  dasatinib I, R | T315I  **G250E**  **L248V** | T315I |  |
| 308 | 300 | 13 | imatinib I, R  nilotinib R | F311L | F311L |  |
| 309 | 600 | 15 | imatinib R  dasatinib I, R  nilotinib I | V299L | V299L | CHR |
| 310 | 300 | 8 | imatinib R  dasatinib I | F359V | Unknown | MMR |
| 312 | 600 | 5 | dasatinib R  imatinib R  nilotinib R  bosutinib R | Absent | Absent |  |
| 313 | 300 | 1.4 | imatinib I  dasatinib I | Absent | Unknown | MR 4.5 |
| 314 | 200 | 15 | imatinib I  nilotinib R | Absent | Unknown | MR 4 |
| 315 | 200 | 3 | imatinib I, R  dasatinib I, R | Absent | Absent | CCyR |
| 316 | 200 | 1.8 | imatinib R  bosutinib I | Absent | Absent |  |

## **Changes to the study protocol or planned analysis**

As of August 9, 2019, four amendments to the original version of the protocol for this clinical trial were issued and approved by the Ministry of Health of the Russian Federation.

Protocol version 2.0 was issued on May 15, 2017 and contained administrative changes to the protocol and the following changes:

- the possibility of expanding the cohort before the establishment of the MTD in the event that DLT was already registered.
- the option to revert to the previous dose of study drug after dose reduction, as well as the option to split the daily dose of study drug into 2 doses in patients with intolerable toxicity.
- a procedure for assessing the total cardiovascular risk using the European SCORE scale.

Protocol version 3.0 was issued on February 21, 2018 and contained administrative and the following changes:

• The number of patients included in the study was increased from 44 to 65.

• Added option to not visit on Day 15 after cycle 4.

• Added the ability to not evaluate cytogenetic response in patients with a complete cytogenetic response and a molecular response of less than 1% BCR-ABL^IS^, and a schedule for assessing the cytogenetic response after the level of BCR-ABL^IS^ exceeds 1%.

• A prohibition to increase the dose of investigational drug above the initially assigned level for cohorts expanded to 10-15 patients, and the procedure for reducing the dose in these cohorts.

Protocol version 4.0 was released on July 31, 2018. Compared to protocol version 3.0, it contained the following changes:

• The conditions for conducting an interim data analysis to report on the main results of the study is introduced.

• Administrative aspects of the study.

Protocol version 5.0 was released on December 27, 2019. Compared to protocol version 4.0, it contained the following changes:

• Information on the composition of finished dosage forms of the drug PF-114 was updated in the Protocol in connection with the addition of excipients to the composition of the finished dosage form (PF-114 mesylate 100 mg in the form of capsules for oral administration) in order to improve its technological characteristics.

• The results of a comparative toxicokinetic study of PF-114 dosage forms with excipients (PF-114 mesylate 100 mg in gelatin capsules with excipients) and without excipients (PF-114 mesylate 100 mg in gelatin capsules) under a single dose administration in dogs are added to Section 6.3 “Results of Pre-clinical studies” of the Protocol.

• In the text of the Protocol (including the title page, synopsis, footers and Section 11.1 “Description of the study drug PF-114”), the name of the drug, finished forms of the study drug and active substance were unified (PF-114 mesylate).

An estimate of the presence of mutations was added based on the results of the sponsor's assessment of three sources of information: medical history, Sanger sequencing and NGS sequencing data. In this assessment, medical history data were not taken into account if they were not confirmed by at least one of the sequencing methods.

## **Bioanalytical Method**

PF-114 was determined in human plasma samples using validated analytical method. The method proved to be precise, accurate, and selective for plasma level determination of PF-114 in the concentration range of 2.0 – 1000.0 ng/mL. The analytical method used 200 μL of plasma sample for each analysis. Analyte was isolated from plasma by a solid-phase extraction procedure performed on Oasis MCX extraction columns.

## **Methods of statistical analysis**

## All study results are presented descriptively by dose level or study as a whole, where appropriate.

Safety Set

Safety set consists of all patients, who at least once took the study drug.

Population for DLT evaluation

Population for DLT evaluation consists of all patients, who received sufficient amount of the study drug during Cycle 1 for adequate DLT assessment.

The sufficient amount of the drug is determined as the intake of ≥ 75 % of the scheduled dose during Cycle 1, except for doses missed due to the DLT.

Population for evaluation of pharmacokinetic properties

According to the Protocol, population for the pharmacokinetic analysis consists of all patients, who took at least one dose of PF-114, with respect to which sufficient amount of data on concentration of the study drug in the blood. The analysis of data on pharmacokinetic properties of the drug is carried out in this population of patients. All PK analyses will be presented on two subsets described in more detail below:

- The PK concentration set (PK-C): This set includes all treated subjects who have at least 1 measured concentration.
- The PK parameter analysis set (PK-P): This set consists of subjects from PK-C, who have no major protocol violation relevant to the evaluation of Pharmacokinetics, and who have at least 1 estimated PK parameter. Listings include all available raw data without any exclusions.
